# Developing a diagnosis model for dry eye disease in dogs using object detection

**DOI:** 10.1038/s41598-022-25867-y

**Published:** 2022-12-09

**Authors:** Joon Young Kim, Myeong Gyun Han, Jae Heon Chun, Eun A. Huh, Suk Jun Lee

**Affiliations:** 1 Department of Veterinary Ophthalmology, College of Veterinary Medicine, Konkuk University, Seoul, 05029 Republic of Korea; 2 KU Center for Animal Blood Medical Science, Konkuk University, Seoul, 05029 Republic of Korea; 3 Division of Business Administration, College of Business, Kwangwoon University, Seoul, 01897 Republic of Korea; 4 AIFORPET Corp, Pohang, Gyeongsangbuk-Do 37673 Republic of Korea

**Keywords:** Zoology, Animal physiology

## Abstract

The purpose of this study was to develop an object detection method for the diagnosis of dry eye disease (DED) in dogs. To this end, a methodology was designed to evaluate ocular surface video images using the YOLOv5 model, which is an object detection algorithm that has been widely used because of its simple network structure and fast detection speed. Because the cornea is a transparent organ, an illuminator plate with grid squares was used to provide grid lines, which were analyzed as the reflected straight lines of the light source representing the precorneal tear film (PTF) stability. The original video consisted of the number of 12 normal images(normal, $n$ = 17) and the number of 15 abnormal images(abnormal, $n$ = 17), converted to JPEG images for labeling, learning, and model validation. The labeled image data were divided into a training image data set (normal, $n$ = 15,276; abnormal, $n$ = 26,196) to a validation image data set (normal, $n$ = 6546; abnormal, $n$ = 11,228). As a result of the experiment, the mean average precision ($mAP$) achieved 0.995. This study proposes a method to effectively determine ocular surface status in dogs by using YOLOv5 and concludes that an object detection model can be used in the veterinary field.

## Introduction

Dry eye disease (DED) is an emerging clinical disorder of the ocular surface tear film causing irritation^1^ of the eye and thus reducing the quality of life^2^. The main diagnostic technique used widely in veterinary medicine is the tear film break-up time (TFBUT) test, which lacks objectivity due to the test being observed by an examiner, and the result is estimated on observation alone. Fluorescein dye is also known to destabilize PTF, affecting the result of TFBUT by the amount of fluorescein dye used^3^. Noninvasive break-up time (NIBUT) using corneal topography with the Placido disc projection technique (concentric ring) is a method commonly used in human medicine and was first described by Mengher et al.^4^ using grid reflection from the corneal surface. While conventional diagnostic tests for DED, such as the Schirmer Tear Test-1 (STT-1) or fluorescein dye TFBUT, lack satisfactory reliability and reproducibility^5^, many of the NIBUT test equipment results in repeatability and correlates with the dry eye symptom score, suggesting good diagnostic value for DED^6,7^.

In the field of veterinary medicine, few studies utilize the NIBUT system to clinically evaluate DED. A pilot study by Kim et al.^8^ reported reference values of dry eye tests in normal beagle dogs using an ocular surface analyzer, addressing the need for additional research.

Deep learning models for image recognition have recently been tested in various fields^9,10,11^. Deep learning is based on an artificial neural network based on the 1990 backpropagation algorithm. An artificial neural network will use the logic of correcting errors in each neuron after analyzing errors in the reverse direction on the output side when errors occur. Artificial neural network models have been stagnant because learning becomes more difficult as the number of layers increases, but recent advances in computer hardware technologies such as high-performance graphics processing units (GPUs) have enabled neural networks to become deep neural networks. Furthermore, the dataset overfitting problem, which has been a persistent problem for artificial neural networks, has been improved using dropout methods. Among the fields of image recognition, detecting an visual object instance in image is called Object detection. Typically, Object detection uses an approach to pre-extract the object feature to be found and detect it within the image. In this study, we used YOLO among various object recognition deep learning algorithms. YOLO is used as Object detection in various fields because it shows superior performance in both the mean average precision ($mAP$) and FPS (Frames Per Second) than existing algorithms such as R-CNN and Fast R-CNN.

With this background, a grid illumination plate is designed to assess the ocular surface film by mounting the plate over a slit-lamp biomicroscope and observing the reflected grid lines over the PTF. A video image is obtained using the slit-lamp biomicroscope for 20 s, which is assessed by a single examiner for the time elapsed since the eye opens to the detection of the first distortion or discontinuity of the grid lines. The evaluation result is used as a label (e.g., normal or abnormal (distorted)) for data used for deep learning model learning. The deep learning model determines whether the eyeball surface state is normal or abnormal, and the performance of the model is determined by the mean average precision ($mAP$) value.

## Results

Corneal video images were obtained from 52 dogs and 95 eyes (49 right eyes and 46 left eyes). The breeds represented in the images were Poodle (14/52), Maltese (12/52), Shih-tzu (9/52), and Bichon Frise (4/52), with other breeds represented the remaining 13/52 images. The mean age of all patients was 8.44 ± 3.35 (mean ± standard deviation (SD)) years. Overall, 29/52 of the dogs were castrated males, 3/52 were intact males, 17/52 were spayed females and 3/52 were intact females.

The maximum number of learning steps for each model was set to 50 to standardize the learning equity. Additionally, the RMSprop optimizer was used as an optimization strategy: the learning rate was set to 0.0001 and the batch size was set to 4.

The precision specifies the accuracy and refers to the ratio of the detection results that are correctly detected.

The recall specifies the detection rate and refers to the ratio of what is predicted to be correct among the results that are actually detected correctly.

$$Precision= \frac{TP(True \,Positive)}{TP+FP(False\, Positive)}$$

$$Recall= \frac{TP}{TP+FN(False\, Negative)}$$

**Figure 1:**
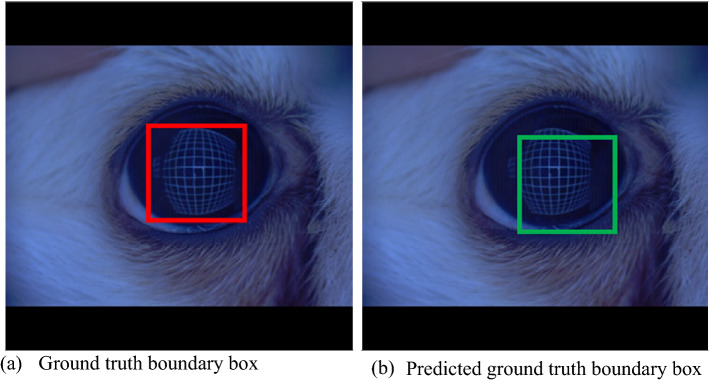
Ground truth boundary box.

As shown in Fig. 1a, ${B}_{gt}$ (the ground truth boundary box) surrounds the object to be detected. Suppose that ${B}_{p}$ (boundary box predicted by the algorithm) is shown in Fig. 1b without the ground truth boundary box.

**Figure 2:**
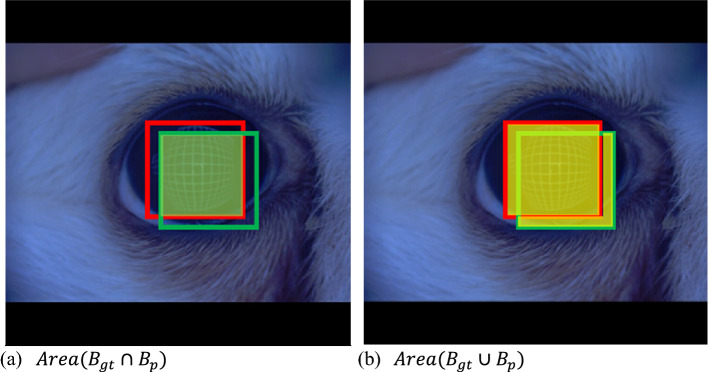
Intersection over union.

In this situation, the intersection over union (IoU) was used to determine whether the predicted detection was correct or wrong. The IoU refers to the area divided by the overlapping part between the predicted boundary box and the ground truth boundary box and the area of the sum of the two boundary boxes (See Fig. 2).

$$IoU=\frac{Area\,({B}_{gt}\cap {B}_{p})}{Area\,({B}_{gt}\cup {B}_{p})}$$

In general, TP and FP are determined based on the IoU value. When the reference value of the IoU is 0.5, it is referred to as $mAP$_50_, and when not otherwise specified, it is calculated as the average value of several IoUs, not as the average value of a single IoU, and the $mAP$ is used in this study, with a 10 IoU threshold value of 0.5:0.05:0.095.

$$mAP= \frac{{mAP}_{0.50}+{mAP}_{0.55}+\cdots +{mAP}_{0.95}}{10}$$

The $mAP$ is the most common metric used to evaluate object detection models. Table 1 represents the $mAP$, precision, and recall values of the experiment.

**Table 1:** Object detection result.

Model	$mAP$_50_	$mAP$	Precision	Recall
YOLOv5	0.995	0.927	0.943	0.995

## Discussion

Dry eye disease (DED) is a common ophthalmic condition causing ocular discomfort. It is by definition, “a multifactorial disease of the ocular surface characterized by a loss of homeostasis of the tear film, and accompanied by ocular symptoms, in which tear film instability and hyperosmolarity, ocular surface inflammation and damage, and neurosensory abnormalities play etiological roles.” In human medicine, the Tear Film & Ocular Surface Society (TFOS) launched a global consensus called Dry Eye Workshop II (DEWS II) to further increase the understanding of DED^12^. In the veterinary field, however, such consensus has not yet been reached, leading to the need for new and updated diagnostic and treatment paradigms for application. One recent study analyzed and reported the tear film status of veterinary patients using the NIBUT system in normal Beagle dogs^8^.

Precorneal tear film (PTF) health is vital to obtaining a clear and healthy ocular surface because it is the first refractive surface of the eye, and lack of its function is diagnosed as either quantitative or qualitative Keratoconjunctivitis Sicca (KCS). Quantitative KCS has been diagnosed using STT-1 or phenol red thread test. On the other hand, many diagnostic tests, including tear film break-up time (TFBUT) and tear osmolarity, are required to diagnose qualitative KCS^2^. However, it is possible that using a traditional qualitative KCS test and a fluorescein dye staining TFBUT test, the results are affected by the amount of fluorescein used. Fluorescein dye is also known to destabilize tear film, hastening PTF break-up. Benzalkonium chloride, a preservative widely used in ophthalmic solution, also destabilizes the tear film. These factors affect the TFBUT results and make it harder to properly diagnose qualitative KCS^3^.

The noninvasive break-up time (NIBUT) test was first documented by Mengher et al.^4^ in human ophthalmology. A spherical grid illuminator is designed to shine reflections on the corneal surface, which are observed to determine the PTF break-up time without applying any ophthalmic solutions, thus the term “noninvasive tear film break-up time”. Although the already commonly distributed slit lamp biomicroscopy could detect the break-up of tear film with magnification and clinical experience, it may lack the precision keratoscopes and corneal topography techniques provide. To further avail its clinical application, the ultimate intention of the study is to suggest a development of an add-on equipment compatible with the slit lamp biomicroscopy that could provide a similarly precise results that advanced ocular surface analyzers produce. Commonly used methods are TFBUT and NIBUT using ocular surface analyzer (OSA, SBM Sistemi). TFBUT is the most routinely used, and easily used by veterinary clinicians because it is not particularly expensive and do not need any more equipment. However, the result evaluation remains subjective among different examiners and the reliability of the test may be called into question. NIBUT using ICP OSA-VET is relatively easy to inspect and it is also good to secure objectivity. However, the equipment is very expensive, and the patient must be examined by another setting of this equipment again after the slit lamp examination has been finished. In order to overcome this inconvenience, we have considered a way to obtain an image using a simple shooting device while performing an ophthalmologic examination on a slit lamp microscope, and based on its results, to utilize artificial intelligence to enhance objectification of NIBUT.

Although human researches report normal values of NIBUT, such data results have not yet been established in the veterinary field, and our work demonstrates the clinical evaluation of the NIBUT integrated into a convolutional neural network (CNN) to improve objectivity. After learning through deep learning, the results were compared with clinical judgment, and foundation data were constructed for use in actual clinical practice. A 20-s video was selected as the duration of examination, for the object detection would require data from both normal findings of healthy subjects and abnormal findings of dry eyes to define “normal” and “abnormal”. It is generally known that the TFBUT of normal dogs are 21.53 ± 7.42 s^13^, 19.96 ± 5.01 s for OD and 19.38 ± 4.80 s for OS^14^. To obtain data representing both the “normal” findings and “abnormal” findings, “20 s” was considered to suffice for the purpose during the experiment design. All the results without tear film break-up were considered “normal”.

All the examinations were performed in a ventilatable, air-conditioned closed room, with basic setting of temperature as described in the methods. To maintain the room settings, the room always maintained ventilated air-conditioning, except when the subject entered the room to be examined. Though the room conditions may affect the test results^15^, a comparative study among different conditions has not been tested, but it is established from human medicine that ventilation is a factor affecting tear film^16^. Uniform room condition was secured to lessen the effects of ambient factors in this study.

Manual opening of eyelids cause faster break-up of tear film. In human medicine, patients are asked to keep the eyelids stay open, which could not be done in veterinary patients. As mentioned in the methods, any video images that failed to capture when tear film spontaneously break up are excluded from the results, such as foreign body floating in air contacting cornea, patients going out of focus, and of course, blinking. The effect of blinking remains enigmatic, for the images including blinking were not included in the analysis. Also, basically, TFBUT test is performed with the eyes compulsorily open for Veterinary Patients. The same applies to veterinary patients in the NIBUT test.

Deep learning is being used in research on breed classification, face identification, and behavioral pattern classification in the field of dogs. The identification of dog breeds is essential for understanding dog health problems and scarlet action behavior. Borwarnginn et al.^17^ proposed a dog breed identification classification model by retraining dog breed data sets and pretrained CNNs, and the proposed model recorded an accuracy of 89%. Nagy and Korondi^18^ proposed a model that recognizes and analyzes actual dog behavior patterns using deep learning to implement dog behavior in robots. The proposed model results show that the implemented neural networks can effectively predict the attention of the dog with 88% accuracy, tail wagging with 82% accuracy, and contact seeking behavior with 88% accuracy. Ferres et al.^19^ defined the emotional state of a dog for a specific pose and proposed a model to predict the emotional state of a dog according to dog pose. Emotional states (anger, fear, happiness, and relaxation) were defined, 100 images of each were learned, and the proposed model classified dog emotions with 62.5% accuracy^20^ conducted a study to classify corneal ulcer severity in dogs using CNNs, a deep learning-based image recognition method. Most of the models proposed in the study achieved over 90% accuracy when classifying corneal and pericardial ulcers. This showed that the severity of corneal ulcers in dogs can be effectively determined using a deep learning model.

## Methods

Data were obtained from 52 patients who visited the Veterinary Medical Teaching Hospital (VMTH) at Konkuk University from August 5th, 2020 to October 1st, 2021. The study was approved by the Institutional Animal Care and Use Committee of Konkuk University (protocol # KU20123), and all dog owners provided written informed consent. The VMTH at Konkuk University, as a routine procedure, requests the owners of all the animals enrolled in the study to fill out a patient consent form, which includes a notification that patient information obtained during treatment may be used for research purposes. This study was conducted in accordance with all applicable regulations and guidelines, and all animals were treated in compliance with the ARVO Statement for the Use of Animals in Ophthalmic and Vision Research.

**Figure 3:**
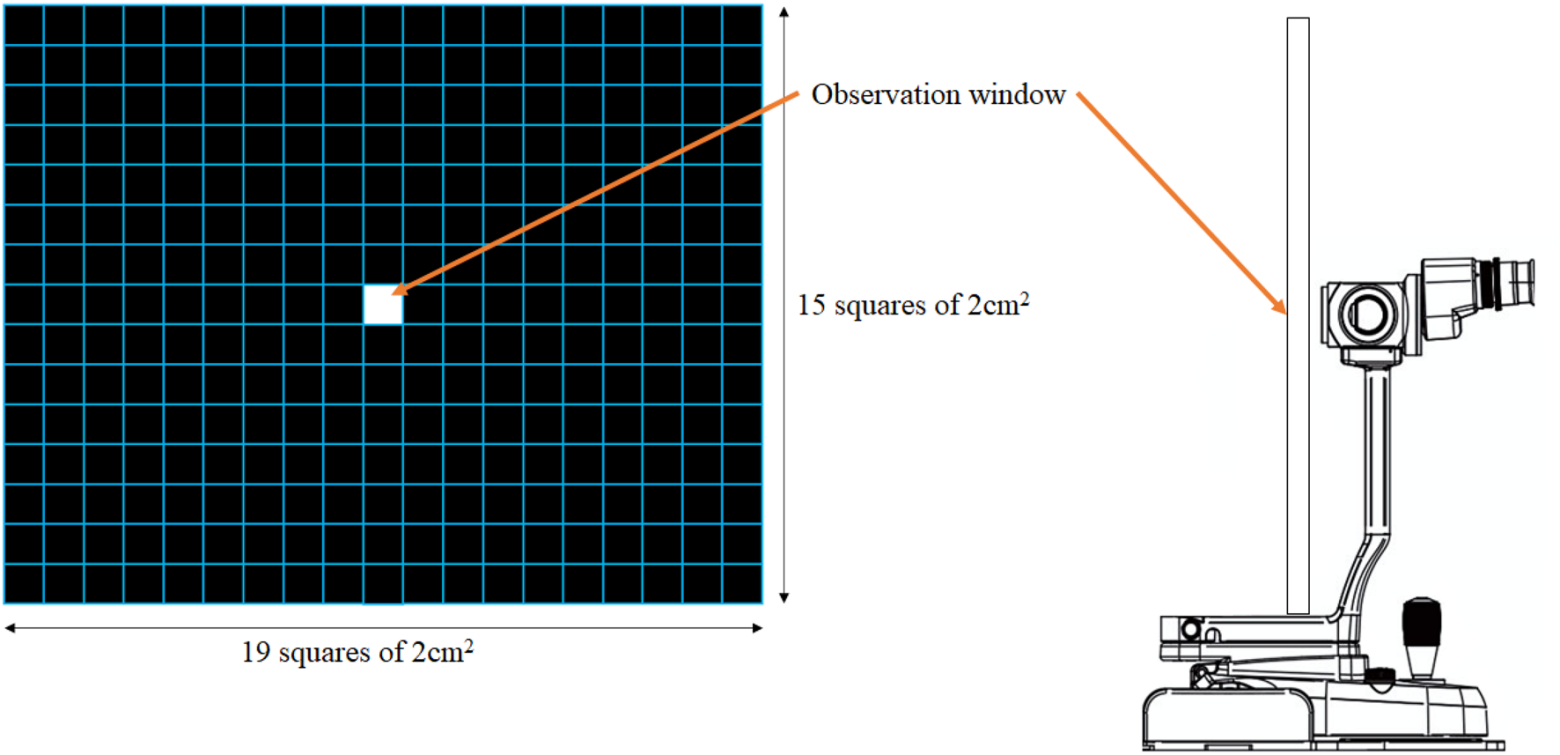
Grid plate design. A window for observation using the slit lamp is located at the center. The plate is placed as close as possible to the slit lamp for maximal visual field from the scope.

A 20-s video image was obtained using a slit-lamp biomicroscope (GS LED Slit Lamp MW50D, Shigiya Machinery Works LTD., Japan) with an custom-made grid-plate illuminator add-on (42 cm by 33 cm in dimension, plate with LED lights emitting from a total of 19 squares by 15 squares, 2 cm^2^ in size grid square lines) from all of the patients (Fig. 3). The video starts as soon as the eyes are opened with gentle force from an assistant restraining the patients to prevent the patients from blinking. We excluded images that failed to cover the entire cornea. All tests were performed in one room with the temperature set between 20 and 25 °C and humidity between 30 and 40%. All ventilations were turned off before the examination began to further reduce factors that may affect the test results.

**Figure 4:**
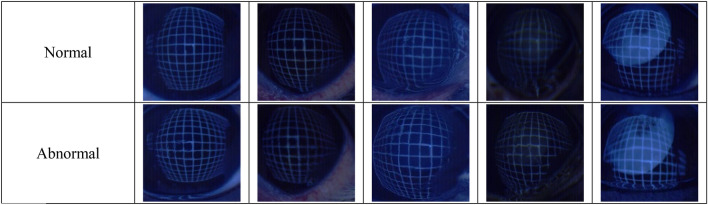
Examples of normal and abnormal images.

Additionally, the sizes of the images differed; the largest was 5184 × 3456 pixels, and the smallest was 1184 × 831 pixels. The largest and smallest cropped images were 2572 × 2672 and 177 × 134 pixels, respectively. The images were resized to 412 × 412 pixels to train the YOLOv5 model. All images were analyzed by a single observer. A count-up timer was started from the start of the video and was stopped as soon as the observer detected any break-up or distortion of the straight grid lines. All imaging data were divided into 4 groups based on the break-up time of the corneal grid lines. Those in which the tear film grid lines broke up in less than 5 s were designated “group 1”, less than 10 s were designated “group 2”, less than 20 s were designated “group 3”, and eyes in which corneal grid lines did not break until the video ended, which was 20 s in duration, were designated “group 4”. Of the 94 eyes, 44 eyes were “group 1”, 17 eyes were “group 2”, 12 eyes were “group 3”, and 22 eyes were “group 4”. Figure 4 shows normal and abnormal images.

The labeled image data were divided into a training image data set (normal, $n$ = 15,276; abnormal, $n$ = 26,196) to be learned by the model and a validation image data set (normal, $n$ = 6546; abnormal, $n$ = 11,228) used for accuracy evaluation based on the learned model.

In this study, the labeled images were used to train the YOLOv5 models. To accomplish this task, we used PyTorch, an open-source software library developed by Facebook’s AI Research lab. The experiment was divided into ”normal” and ”abnormal” to assess the performances of the models. The models were fine-tuned using the weights of the fully connected layer starting with a YOLOv5 model trained by the MS COCO dataset and then used to predict severity. For this purpose, we obtained the YOLOv5 model code from GitHub (https://github.com/ultralytics/yolov5).

## Object detection

Object detection is a computer vision task that deals with detecting a visual object instance in an image^21^. In computer vision, three terms are used interchangeably: object detection, object recognition, and object tracking^22^. Object recognition means distinguishing what an object is like, and object detection determines only the existence of a smaller range of objects than recognition. In other words, to perform object recognition, object detection must precede object detection because it is a matter of finding an object in the image and what it is.

Usually, object detection uses an approach that preextracts features of the object you want to find and detects them within a given image. After extracting features, an algorithm determines the boundary from the distribution of features detects objects by using the same detection algorithm, such a support vector machine (SVM) and adaptive boosting (Adaboost), to distinguish which features represent objects or not represent an object. In other words, the object detection algorithm follows a pipeline of preprocessing, feature extraction, and classification. Recently, various detection and recording algorithms, such as the region based CNN (R-CNN), Fast R-CNN, and you only look once (YOLO) based on convolutional neural networks, have been developed during deep learning. The object detection algorithm in the deep learning algorithm integrates and processes object detection and recognition, and the YOLOv5^23^ algorithm was used in this study. YOLOv5 is a model that performs better in both the $mAP$ and frames per second (FPS) than the conventional R-CNN and Fast R-CNN and is used as an object detection algorithm in various fields^20^.

**Figure 5:**
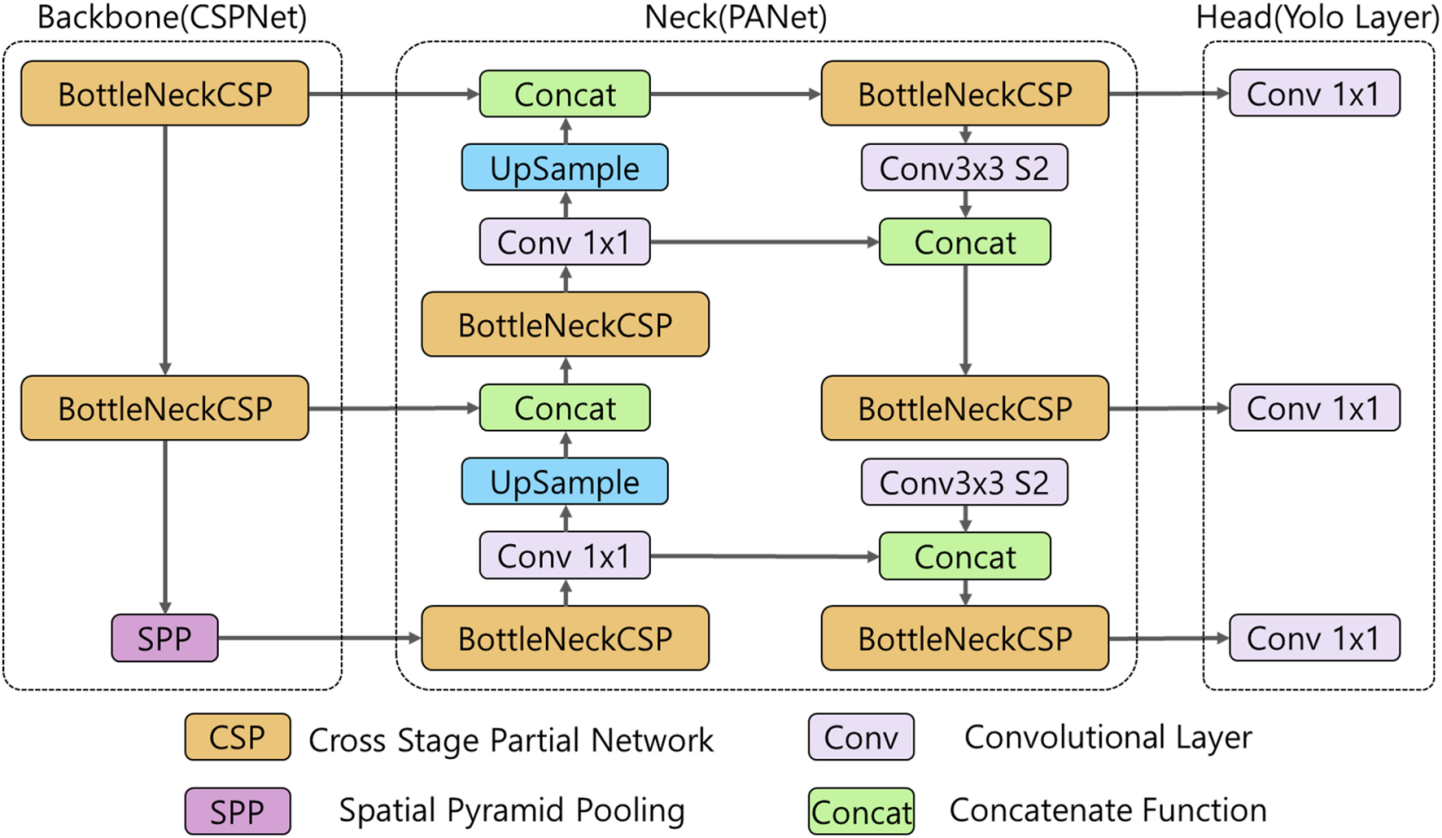
The network architecture of YOLOv5^26^.

As shown in Fig. 5, YOLOv5 consists of a model backbone, model neck, and model head, similar to other object detectors. The model backbone is used to extract important features from a given input image. In YOLOv5, a cross-stage partial network (CSPNet)^24^ is used as a model backbone to extract rich information from input images. The model neck is used to generate feature pyramids that help the model generalize object scaling well. It helps to identify the same object of different scales and sizes. In YOLOv5, a path aggregation network (PANet)^25^ is used as a model neck to obtain feature pyramids. The model head is used to apply anchor boxes on features and generate final output vectors with class probabilities, objectiveness score which is the probability value of whether it is an object or not scores, and bounding boxes. The model head is used to generate three size feature maps (18 × 18, 36 × 36, and 72 × 72) to achieve multiple scale predictions, allowing the model to handle small, medium and large objects^26^.

## Conclusions

Corneal reflection video images of the canine ocular surface using a grid plate illuminator were analyzed to assess corneal tear film stability without invasive ophthalmic solution application.

**Figure 6:**
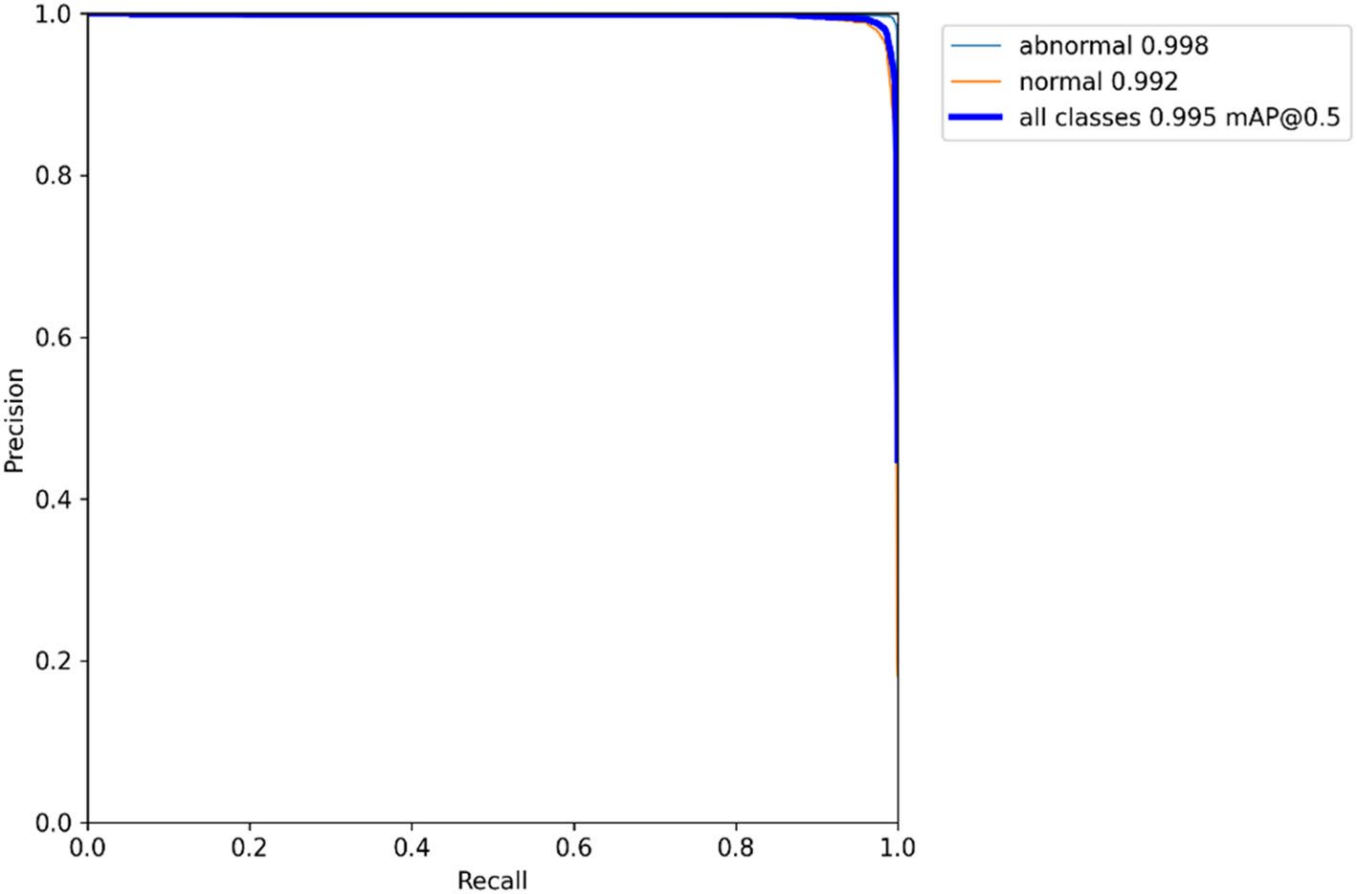
Precision-recall curve.

To teach a model to classify whether corneal reflection on the surface of the eye is normal, 43 images available among 52 subjects of corneal tear film were used as learning data for deep learning models. The empirical analysis results of this study showed that YOLOv5 exceeded 0.955 $mAP$_50_ 0.995, precision 0.943, and recall 0.995(See Fig. 6). High performance was achieved using high-quality image data in this work. If a significant portion of an image is not actually available or is degraded in clinical practice, the usefulness of the image in actual clinical applications may be limited. The limitations of this study are primarily in evaluating manual corneal tear film stability.

Further investigation using noninvasive methods is required to set standardized references for normal values in the veterinary field, and this study suggests a way to assess precorneal tear film stability using a grid plate illuminator combined with a convolutional neural network system, a promising technology to assess images objectively.

## Data Availability

The datasets generated and/or analysed during the current study are available in the [public] repository, [https://drive.google.com/file/d/1xm-v4I_kBo8LgGL_UUVc3Yrnhsg1ztW-/view?usp=share_link].
